# The Prevalence of Mycobacterium tuberculosis Infection Among Cancer Patients Receiving Chemotherapy in a Tertiary Care Center

**DOI:** 10.7759/cureus.32068

**Published:** 2022-11-30

**Authors:** Mona A Aldabbagh, Asma Abughasham, Ghaday Alansari, Suaad Bougis, Ealaf Melibari, Nada Alhatmi, Muhammad A Khan, Wasil Jastaniah

**Affiliations:** 1 Department of Pediatrics, King Abdulaziz Medical City, Ministry of National Guard Health Affairs, Jeddah, SAU; 2 Medicine, King Saud Bin Abdulaziz University for Health Sciences College of Medicine, Jeddah, SAU; 3 Medical Education, King Saud Bin Abdulaziz University for Health Sciences College of Medicine, Jeddah, SAU; 4 Research, King Abdullah International Medical Research Center, Jeddah, SAU; 5 College of Medicine, King Abdulaziz Medical City, Jeddah, SAU; 6 Department of Oncology, King Faisal Specialist Hospital and Research Centre, Jeddah, SAU

**Keywords:** interferon-gamma release assay (igra), immunosuppression, cancer, chemotherapy, mycobacterium tuberculosis

## Abstract

Objective: To estimate the prevalence of tuberculosis (TB) infection among patients receiving cancer chemotherapy and to identify risk factors for latent TB reactivation.

Methods: A cross-sectional study was conducted at a tertiary care center in Jeddah, Saudi Arabia. Patients were surveyed for TB risk factors, their records were reviewed for previous TB infection or disease, and blood samples were collected for interferon-gamma release assays (IGRAs).

Results: A total of 203 patients were included. One hundred and twenty-five were females (62%). Median age was 52 years, and mean age was significantly higher in positive IGRA patients compared to negative IGRA (57.32 vs. 47.27; *p *= 0.009). Twenty-five patients (12.3%) had evidence of TB infection, 16 (68%) among them had a latent TB infection, while the rest received treatment for an active TB disease. The rate of active disease among cancer patients was 8 (3.9%). Additionally, 92% (23) of those with positive IGRA had solid cancers (*p *= 0.007), and all active TB cases occurred in this group of solid cancers.

Conclusion: TB prevalence was higher in chemotherapy patients compared to the general Saudi population. Patients with solid tumors and older age had a greater risk of developing the infection, signifying the importance of preventing TB and malignancy coexistence by initiating screening policies in cancer patients.

## Introduction

Tuberculosis (TB) is a major public health issue worldwide. In 2018, it was estimated that 10 million individuals have been diagnosed with TB globally, resulting in a total of 1.5 million deaths that year [1]. In Saudi Arabia, a total of 64,345 new TB cases were reported over a 20-year period, and TB was rated number 11 among the top leading causes of death in the kingdom according to the World Health Organization (WHO) [1,2]. In addition, the total incidence of TB in 2018 was 10 cases per 100,000 people [2]. A study in 2013 showed that the western region had a two-fold higher incidence than the national TB incidence level (24/100,000) due to incoming pilgrims [3]. Another recent study conducted at the Ministry of National Guard Health Affairs in Riyadh, Jeddah, Al Ahsa, and Dammam, reported the prevalence of latent TB infection to being 9.1% [4].

TB is an airborne infectious disease caused by *Mycobacterium tuberculosis* (MTB) which mainly affects the lungs; however, it can spread to several other organs. TB is asymptomatic initially if the bacteria is dormant and the immune system is competent, causing latent TB infection (LTBI). Later, the disease might reactivate should the immunity be suppressed for any reason. Patients with LTBI who are living in an endemic area, undergoing renal dialysis, had organ transplantation or receiving anti-cancer agents such as tumor necrosis factor-alpha blockers (TNF-α blockers) drugs are at a significantly higher risk for reactivation of the infection [5].

Chemotherapy, which is a combination of aggressive drugs used to cure malignancies by inhibiting the cell’s proliferation or inducing apoptosis, targets the malignant fast-dividing cells, but it can also affect many other normally dividing tissues such as the bone marrow, resulting in a reduced white blood cell count and weakened immune responses [6].

A study performed at Tygerberg hospital in Cape Town showed that the prevalence of TB among pediatric cancer patients was 22 times higher compared to children from a matching age group and environment [7]. Another study was done with a total of 213 patients suffering from various hematological malignancies, and 34 patients developed TB with a total incidence of 16% over the course of the study period [8]. Additionally, according to research that was performed in Istanbul, out of the 70 lymphoma cancer patients, 14 (20%) had developed TB during the treatment. However, a definitive diagnosis was difficult to make because the clinical presentations were almost identical to those of lymphoma, and the tuberculin skin test (TST) results were altered by the impairment of the cellular immune system [9].

The diagnosis of TB has been proven difficult because it requires clinical, microbiological, serological, and radiological screening to confirm the presence of active bacteria. The main two methods used to screen for LTBI are the TST and interferon-γ release assays (IGRAs). TST also has limitations in specificity, where it can give false-negative results in immunocompromised patients and false-positive results in children who have received the Bacillus Calmette-Guerin (BCG) vaccine. On the other hand, the IGRA test has proven to have a higher specificity for MTB [10]. However, both tests cannot differentiate LTBI from the active form of the disease. Chemotherapy can make it even more challenging to detect TB due to the bacterium’s virulence factors and the limitations of diagnostic tools [11].

A recent study conducted at the Ministry of National Guard Health Affairs in Riyadh, Jeddah, Al Ahsa, and Dammam, reported the prevalence of LTBI to be 9.1% [4]. However, this study did not look in depth at the rate of LTBI in chemotherapy patients. This study was designed to estimate the prevalence of LTBI among cancer patients receiving chemotherapeutic agents and are vulnerable to TB activation, in the tertiary care center of King Abdulaziz Medical Center-National Guard Hospital Affairs-Jeddah. Furthermore, the study aims to examine the effect of sociodemographic factors on the prevalence of the infection and activation and to identify the risk factors associated with the progression to active disease. Knowing this information will assess the need for developing TB screening policies in this high-risk population prior to the initiation of chemotherapy to prevent disease reactivation.

## Materials and methods

This is a cross-sectional study conducted at King Abdulaziz Medical City (KAMC), a tertiary care center in Jeddah, Saudi Arabia between June 2020 and March 2021. This study was approved by the Institutional Review Board of King Abdullah International Medical Research Center (Approval number: SP20/048/J).

The participants were selected consecutively from the oncology department which has 1500 new cases per year. Patient demographics, including gender, age, and type of cancer they are receiving the chemotherapy for were collected from electronic medical records.

All participants were interviewed via phone. Subjects with documented IGRA results in the medical records were contacted and surveyed for risk factors for TB and any past exposure to the bacteria after a consent form was explained and taken in the presence of two witnesses. Patients who were eligible for the study but had no available IGRA results were interviewed and asked to visit the hospital and give a blood sample to be tested.

The included subjects were any cancer patients receiving chemotherapy during the study period, and the study included both adults and children. While patients who ended their chemotherapy course before the designated date, patients who would not agree to give a blood sample for the IGRA test, and patients undergoing chemotherapy for other reasons than malignancies were excluded.

Using the Raosoft software sample size calculator and given a known population size of 1500 patients admitted to Princess Noorah Oncology Center -Jeddah annually, and using a margin of error of 2.3%, derived from the 9% reported prevalence of TB infection, and a confidence level of 95%, the desired sample size was estimated to be 822 [4].

The questionnaire was made by the authors, and it was validated by four validators: two infectious diseases physicians to validate the construct and content validity, and two from the medical education department for face validity. The questionnaire was composed of 13 questions and addressed the sociodemographic background in addition to questions about the history of TB exposure, disease history, and risk factors. Information such as IGRA results, treatment, and type of cancer was obtained from the electronic medical records. Translation to Arabic was done by the co-authors, evaluated during the validation process, and further assessed using a pilot study.

For data analysis, numbers and percentages were used to describe categorical variables, while means and standard deviation were used for continuous data. The Chi-square or Fisher’s exact test are used as appropriate to compare categorical data and a p-value cutoff of less than 0.05 was considered statistically significant. The data was analyzed using the Statistical Package for Social Sciences (SPSS)-Version 20.0.

## Results

Out of 1400 patients contacted, 313 agreed to be included in the study, making a response rate of 22%. However, 110 were later excluded from the study afterward because of the unattainability of blood samples. So, a total of 203 patients’ data was used for the final analysis. One hundred and twenty-five of the cases were females (62%) and 78 were males (38%). The median age was 52 years (range, 38-62 years). Moreover, most patients were living in the Makkah region with a total of 139 (68%). In all included subjects, 25 had positive IGRA results (13%), 170 had negative results (83.7%), and eight had indeterminate IGRA results (3.9%) and were further excluded from the analysis; no one from this group, however, developed active TB disease. As shown in Table 1, cancer patients with positive IGRA were significantly older than those in the negative IGRA group (57.32 years vs. 47.27; *p* = 0.009). Furthermore, IGRA positivity was significantly higher in people who reported a history of TB contact and previous TB disease (*p* = 0.022). In addition, there was a significant difference in IGRA positivity based on cancer type, where IGRA-positive results were significantly higher in patients with solid tumors, compared to those with hematological malignancies (92% vs. 8%; *p* = 007).

**Table 1:** Demographic features of all included subjects classified by IGRA results *Chi-squared test **Fisher exact test ***19 of the patients were children under 18 years of age, and all of them had negative IGRA IGRA: interferon-gamma release assays

IGRA results				
		Negative	Positive	p-value
		n = 170	n = 25	
Age ***(Mean +/-SD)		47.27 (18.21)	57.32 (13.85)	0.009*
		n (%)	n (%)	
Sex (%)	Female	105 (61.8)	17 (68)	0.548*
	Male	65 (38.2)	8 (32)	
Marital status (%)	Married	100 (58.8)	17 (68)	0.066**
	Single	36 (21.2)	1 (4)	
	Divorced	14 (8.2)	1 (4)	
	Widower	20 (11.8)	6 (24)	
Type of cancer (%)	Solid	111 (65.3)	23 (92)	0.007*
	Hematological	59 (34.7)	2 (8)	
Current region of residence	Al Bahah	8 (4.7)	1 (4)	0.288**
(%)	Aseer	8 (4.7)	1 (4)	
	Makkah	121 (71.2)	18 (72)	
	Hail	3 (1.8)	0 (0)	
	Riyadh	1 (0.6)	0 (0)	
	Madinah	28 (16.5)	3 (12)	
	Jazan	1 (0.6)	1 (4)	
	Eastren	0 (0)	1 (4)	
Previously diagnosed with TB (%)		1 (0.6)	8 (32)	<0.001**
Treated from TB (%)		1 (33.3)	8 (100)	0.055**
History of TB contact (%)		9 (5.3)	5 (20)	0.022**

As shown in Table 2, 17 (68%) of the patients with positive IGRA had LTBI, and eight (32%) had active disease with chest X-ray findings suggestive of pulmonary TB, making the rate of active disease among cancer patients around 3.9%. Furthermore, anti-TB treatment for the active disease was documented for only nine patients of the 25 patients who had positive results. All patients who were diagnosed with active TB infection (100%) had solid cancer, specifically breast cancer which was the most common solid tumor in our sample and the most reported tumor among the TB-diseased cases (38%), however, this did not reach statistical significance (data not presented). In addition, patients diagnosed with active TB infection had a longer duration of chemotherapy compared to LTBI; however, this did not reach statistical significance (*p* = 0.060). Most participants were from the Makkah region followed by Madinah, Aseer, and Al Bahah, respectively. Out of the 25 patients with positive results, eight (32%) reported traveling outside of Saudi Arabia. Patients who were diagnosed with TB did not report any visits to prison or nursing homes. Additionally, none of the diseased patients reported symptoms such as coughing, hemoptysis, fever, or night sweats.

**Table 2:** Comparison between active vs. latent TB among IGRA positive subjects *Independent t-test **Fisher exact test IGRA: interferon-gamma release assays

		Latent TB	Active TB	p-value
Age		Mean (SD)	Mean (SD)	
		58.2 (10.65)	55.5 (19.81)	0.168*
		n (%)	n (%)	
Sex (%)	Female	12 (0.706)	5 (0.625)	0.513**
	Male	5 (0.294)	3 (0.375)	
Marital status (%)	Married	12 (0.7)	5 (0.625)	0.606**
	Single	1 (0.059)	0 (0)	
	Divorced	0 (0)	1 (0.125)	
	Widower	4 (0.235)	2 (0.25)	
Type of cancer (%)	Hematological cancer	2 (0.118)	0 (0)	0.453**
	Solid tumor	15 (0.882)	8 (1)	
Region of residence (%)	Makkah	11 (0.647)	7 (0.874)	
	Madinah	3 (0.176)	0 (0)	
	Aseer	0 (0)	1 (0.125)	
	Al Bahah	1 (0.059)	0 (0)	
Previously diagnosed with TB (%)		4 (0.235)	4 (0.5)	0.359**
History of TB contact (%)		3 (0.176)	2 (0.25)	0.435**
Referral to ID department (%)	Referral	4 (0.235)	5 (0.625)	0.087**
	No referral	13 (0.765)	3 (0.375)	
Duration of TB therapy (%)	3 months	0 (0)	1 (0.25)	–
	6 months	0 (0)	2 (0.5)	
	>9 months	0 (0)	1 (0.25)	
Chemotherapy duration currently (%)	<3 months	1 (0.059)	1 (0.125)	0.060**
	3 to 6 months	4 (0.235)	0 (0)	
	>6 months	7 (0.412)	7 (0.875)	
	Can’t remember	5 (0.294)	0 (0)	
Received chemotherapy in the past (%)	Received chemotherapy in the past	9 (0.529)	2 (0.25)	0.234**
	First time	8 (0.471)	6 (0.75)	

## Discussion

TB is a major health issue in Saudi Arabia. Patients with LTBI who are undergoing dialysis, organ transplantation, or anti-cancer treatment are known to have a higher risk of reactivation of the infection [5]. This study assessed the prevalence of LTBI among cancer patients receiving chemotherapeutic agents and who are vulnerable to TB activation in a tertiary care center in Jeddah, Saudi Arabia. The study found that the prevalence of TB infection among cancer patients receiving chemotherapy is 13%, while TB disease occurred in 3.9%. In addition, TB infection was more among older age groups and significantly higher in those with solid tumors.

Our data showed significantly more positive IGRA results among the older age group, as it is well known that advanced age is an important risk factor for the reactivation of TB infection since, with advanced age, the immune system changes leading to increased susceptibility to infectious diseases [12]. DNA damage, protein misfolding, and decreased cell function at the cellular and molecular levels may explain the possible mechanisms of immune system impairment in the elderly [13,14]. Furthermore, the risk of having TB is accumulative throughout life [15].

In the general population of Saudi Arabia, a study reported the prevalence of TB infection to being 9.1%, which was lower than the prevalence of TB among cancer patients in our study [4]. This could be justified by the fact that the study was performed in multiple regions of Saudi Arabia, and our study was concentrated in the western region. In addition, the mean age of included cases in our study is older than that study [4].

It is recognized by the Center for Disease Control and Prevention and the American Thoracic Society that cancer is a risk factor for the development of TB infection, and many other studies that the prevalence of TB in cancer patients was higher than in the general population [16-18]‏. One systematic review stated a nine-fold increased risk of developing active TB in patients with hematologic, head and neck, and lung cancers compared to those with no cancer patients [19]. Furthermore, a study investigating the incidence of active TB among pediatric cancer patients, mostly acute lymphoblastic leukemia, showed a 22 times higher rate of the infection than the overall TB incidence reported in children from a similar background, and more importantly, 47% of the active infections appeared in the first five months of chemotherapy, suggesting a reactivation of a latent TB [7]. The proposed mechanism for the infection is likely due to intrinsic immunosuppression of cancer, immunosuppressive effects of chemotherapy, or other host factors that increase the susceptibility to both TB and cancer resulted by a decrease in local infection barriers, leading to the inability to eliminate the infection [16]. This study reported that 3.9% of the included cases during the study period had active TB disease, all of them had solid tumors while none had hematologic malignancy. A study by Kim et al. reported that patients with solid-organ malignancies had a 4.7 times greater risk of having active TB compared to healthy individuals [20]. Our data showed more positive IGRA results and a higher rate of disease activation among patients who had solid tumors, which is also comparable with a case series of 24 patients with active TB, of whom, 23 had solid tumors [21]. On the other hand, a meta-analysis supported that all types of cancer increased the risk of active TB infection, with variation in the cumulative incidence rate/100,000 population (CIR) stratified by cancer type and TB incidence, where the CIR in low TB incidence countries being highest in hematological malignancies (418/100,000 population), while that reported in solid cancers was 244/100,000 population [19]. One study done in Qatar concluded that among 215 subjects with acute myelocytic leukemia identified during the study period, 12 (5.58%) were diagnosed with TB, in comparison to our study which reported no cases in those with hematological malignancies [22]. This could be justified by the actual higher number of included solid tumor cases in this study compared to the cases with hematological malignancies.

Reactivation of a latent TB has been linked to certain chemotherapeutic agents; it is accepted that immune checkpoint inhibitors (ICIs), which are used to treat many cancers such as metastatic melanoma, non-small lung cancer, and head and neck squamous carcinomas, may cause severe infections like reactivation of LTBI indirectly due to using corticosteroids or TNF-α inhibitors. However, TB reactivation may present as a direct complication of ICIs alone, as most reported cases did not receive corticosteroids or TNF-α inhibitors when their reactivation was detected [23]. Additionally, a retrospective cohort study showed that the incidence of TB was eightfold higher in the patients who took ICIs compared to the general population. However, there were no statistically significant differences in the risk of TB in cancer patients based on ICI exposure. Therefore, the high rate of TB in the group who took ICIs in their study was likely due to underlying malignancy since most patients had lung cancer. In addition, the concomitant use of immunosuppressive agents to control immune-related adverse events may also contribute to the development of TB in those patients [24]. Other medications that had been linked to TB reactivation are doublet platinum-based chemotherapy, which are a ministry of drug treatment for non-small cell lung cancers [16].

Furthermore, a meta-analysis of 13 studies with more than 920,000 patients estimated the incidence rate ratio (IRR) of TB active disease among patients with solid and hematologic cancers compared to the general population. The study found that lung, gastric, breast, and colon cancers had statistically significant higher IRR of TB infection. Nevertheless, the IRR for active TB patients with hematologic tumors was greater than for solid tumors [18]. Similarly, according to a large cohort study with a population of 495,335, most patients who acquired TB disease after being diagnosed with cancer were the ones who had hematological type especially lymphoma and Myeloproliferative neoplasms [25]. This is in contrast to a prospective cohort study that found that eight out of 141 patients had high-grade non-Hodgkin’s lymphoma and had a previous history of latent TB. None of the patients developed reactivation of TB when they were treated with cyclical cytotoxic chemotherapy [26]. In our results, 100% of cases of active TB had a solid tumor, and the most common type of malignancy among patients with active TB disease was breast cancer. Similarly, breast cancer was the most common type of malignancy among patients with positive IGRA. This can be since the chance that the sample selected from the oncology patients were mostly females suffering from breast cancer. In contrast to solid tumors, hematological cancer patients had less positive IGRA results and TB disease activation in our study.

The Centers for Disease Control and Prevention (CDC) identify persons with some hematologic disorders such as leukemias and lymphomas and other specific malignancies such as carcinoma of the head or neck and lung as high risk, and they recommend the consideration of the treatment of LTBI in these groups [27]. Routine screening for LTBI in cancer patients and the exclusion of active disease before the initiation of cancer therapy might be needed in TB-endemic areas, and the decisions to screen patients should be based on the recent epidemiologic data locally with an appropriate follow-up, evaluation, and management of positive cases, to prevent TB infection or disease coexistence with malignancy, and to decrease the TB burden in general.

Several limitations were encountered while conducting the study. The data collection was delayed many times due to the COVID-19 pandemic lockdown since patients had limited access to the hospital during the pandemic and could not have the test done accordingly. Moreover, the sample size was small because of the poor response rate, mainly in providing blood samples; this was also related to the limited healthcare access during the pandemic. This resulted in selection bias during the data collection and recruiting patients who had already documented IGRA results available. Moreover, the results are not generalizable as the sample included only patients who received chemotherapy at the National Guard Hospital in Jeddah. The available data on the stage and grade of cancer was limited, which restricted assessing their impact on TB progression. Our recommendation for further studies is to be widely generalized on a multicenter scale across the country with more subjects with hematological malignancies. The inclusion of pediatric cases would also be worth consideration to assess their risk of TB disease in the settings of BCG vaccination. Such information would guide us in initiating TB screening and preventive strategies for high-risk groups.

## Conclusions

TB infection prevalence was higher in chemotherapy patients compared to the general Saudi population. Solid tumors and older age groups had higher rates of positive IGRA compared to hematological cancers and younger patients and TB activation occurred primarily in those with solid tumors, which signifies the importance of preventing the coexistence of TB and malignancy by developing screening programs in cancer patients who are at higher risk for TB activation.
